# Increase in Red Blood Cell-Nitric Oxide Synthase Dependent Nitric Oxide Production during Red Blood Cell Aging in Health and Disease: A Study on Age Dependent Changes of Rheologic and Enzymatic Properties in Red Blood Cells

**DOI:** 10.1371/journal.pone.0125206

**Published:** 2015-04-22

**Authors:** Daniel Alexander Bizjak, Christian Brinkmann, Wilhelm Bloch, Marijke Grau

**Affiliations:** Department of Molecular and Cellular Sport Medicine, German Sport University Cologne, Cologne, Germany; University Medical Center Utrecht, NETHERLANDS

## Abstract

**Aim:**

To investigate RBC-NOS dependent NO signaling during *in vivo* RBC aging in health and disease.

**Method:**

RBC from fifteen healthy volunteers (HC) and four patients with type 2 diabetes mellitus (DM) were separated in seven subpopulations by Percoll density gradient centrifugation.

**Results:**

The proportion of old RBC was significantly higher in DM compared to HC. In both groups, *in vivo* aging was marked by changes in RBC shape and decreased cell volume. RBC nitrite, as marker for NO, was higher in DM and increased in both HC and DM during aging. RBC deformability was lower in DM and significantly decreased in old compared to young RBC in both HC and DM. RBC-NOS Serine^1177^ phosphorylation, indicating enzyme activation, increased during aging in both HC and DM. Arginase I activity remained unchanged during aging in HC. In DM, arginase I activity was significantly higher in young RBC compared to HC but decreased during aging. In HC, concentration of L-arginine, the substrate of RBC-NOS and arginase I, significantly dropped from young to old RBC. In DM, L-arginine concentration was significantly higher in young RBC compared to HC and significantly decreased during aging. In blood from healthy subjects, RBC-NOS activation was additionally inhibited by N^5^-(1-iminoethyl)-L-Ornithine dihydrochloride which decreased RBC nitrite, and impaired RBC deformability of all but the oldest RBC subpopulation.

**Conclusion:**

This study first-time showed highest RBC-NOS activation and NO production in old RBC, possibly to counteract the negative impact of cell shrinkage on RBC deformability. This was even more pronounced in DM. It is further suggested that highly produced NO only insufficiently affects cell function of old RBC maybe because of isolated RBC-NOS in old RBC thus decreasing NO bioavailability. Thus, increasing NO availability may improve RBC function and may extend cell life span in old RBC.

## Introduction

40–50% of the human blood consists of red blood cells (RBC). The daily turnover of an adult amounts to 200 billion RBC per day to maintain the total number of 20–30 trillion RBC [1]. Thus, RBC are considered as a heterogeneous population with cells of different age. The mean RBC life span of healthy subjects is about 115–120 days [2,3]. In contrast, RBC from patients with type 2 diabetes mellitus show a reduced RBC life span [4]. RBC experience morphological [5,6] and physiochemical [7,8] changes during their life time in the human circulation. Membrane microvesiculation of RBC to discard oxidative waste products with simultaneous loss of cytosolic components leads to increased density and rigidity of aged RBC [9,10]. Among others, these changes induce senescence, phosphatidylserine (PS) externalisation [11] and clearance signals to remove the RBC from the cardiovascular system by the reticuloendothelial / macrophage system.

RBC enzymatically produce nitric oxide (NO) by a functional RBC- nitric oxide synthase (RBC-NOS) [12]. Nitric oxide is a key regulatory molecule with extensive metabolic, vascular, and cellular effects [13,14]. NO synthesis is reduced in subjects with type 2 diabetes mellitus [15] and associated with endothelial dysfunction and diabetic vascular complications including atherosclerosis and other vascular pathologies [16].

So far, [17–22] it was presumed that in RBC all possible vasoactive produced NO has to be scavenged by omnipresent hemoglobin. However, multiple reaction routes have been described for NO. NO binding to hemoglobin either results in formation of S-nitrosated hemoglobin (SNO-Hb) or iron nitrosyl hemoglobin (HbNO). Thus, the former is mainly found in oxygenated arterial blood while the latter is prevalent in deoxygenated venous blood [23–26]. NO dissociates from the hemes with faster dissociation rates found when hemoglobin is in the T-state than in the R-state [27,28]. It was also reported that reaction of NO with oxyhemoglobin results in the formation of methemoglobin and nitrate [23]. NO also reacts with molecular oxygen to form nitrite [29] and recent data suggest nitrite as the primary NO storage molecule [23,30] that can be reduced to bioactive NO by deoxygenated hemoglobin [31,32] and deoxygenated myoglobin in hypoxic conditions [33] to mediate a spectrum of physiological responses in blood and tissue [34] including hypoxic vasodilation [35].

Recently, another reaction route of NO within RBC was described. Increased RBC-NOS dependent NO synthesis was shown to increase S-Nitrosylation of cytoskeletal α- and β-spectrins, which was directly linked to increased RBC deformability [36]. Other studies confirm a positive effect of NO on RBC deformability [12,37–40]. Reduced RBC deformability was reported for patients with type 2 diabetes mellitus [41]. Deformability of RBC is important in order to enable the passage of the RBC through the microcirculation and to supply oxygen to the surrounding tissue. It has been suggested that the impaired perfusion at the tissue level observed as a complication of diabetes is primarily due to the reduced RBC deformability [42,43]. Thus, the microcirculation is directly influenced by intracellular NO availability [36].

Arginase, a key enzyme of the urea cycle, is a main regulator and competitor for RBC-NOS activity [44,45] Arginase I is expressed in a variety of cells [46] including RBC [47] and increased arginase expression and activity have been described in animal models [48,49] of diabetes mellitus and in diabetic tissue in humans, as well [50]. Both, arginase I and RBC-NOS compete for their common substrate L-arginine. This mechanism may be of importance for reduced NO availability and endothelial dysfunction associated with type 2 diabetes mellitus. Inhibition of arginase I has been shown to increase NO content in RBC and to protect from myocardial ischemia-reperfusion injury [44]. In patients with type 2 diabetes mellitus, arginase inhibition improved endothelial function [51].

Because changes in RBC-NOS dependent NO production and cell function during *in vivo* RBC aging are unknown, the present study aimed to investigate arginase I activity, RBC-NOS activation, nitrite concentration, reflecting NO production, and deformability of RBC during *in vivo* aging in healthy subjects and patients with type 2 diabetes mellitus. We prove the question whether RBC-NOS and arginase I compete for their common substrate during aging and whether RBC-NOS represents a key enzyme being involved in the aging process. The results of the study shall contribute to the development of new strategies to reduce the cardiovascular complications described for diseases accompanied with decreased RBC life span like diabetes mellitus.

## Materials and Methods

### Ethical approval

The protocols used in this study were approved by the ethics committee of the German Sport University Cologne. These protocols align with the Declaration of Helsinki and all participants gave written informed consent to participate in this study.

### Selection of Subjects

Fifteen healthy subjects and four untrained, non insulin dependent patients with type 2 diabetes mellitus participated in this study. Basal anthropometric parameters of healthy subjects (HC) were as follows (mean ± SD): age [years]: 27.0 ± 5.4, height [cm]: 175.0 ± 10, weight [kg]: 73.1 ± 12.3. Basal anthropometric parameters of type 2 diabetics (DM) were as follows (mean ± SD): age [years]: 52.3 ± 2.6, height [cm]: 178.5 ± 5, weight [kg]: 121.9 ± 19.8.

### Sample Preparation

Blood was sampled from the *Vena mediana cubiti* of fasting human volunteers and anticoagulated using Heparin vacutainers (BD Vacutainer, USA). In total, 16 ml of venous blood was taken, unified in a sterile centrifugation tube and centrifuged at 3000 rcf for 1 min at 4°C. After centrifugation, plasma und buffy coat were removed and isolated RBC were washed with nine fold volume of GASP-buffer (isotonic buffer containing albumin and glucose: 9 mmol/L Na_2_HPO_4_, 1.3 mmol/L NaH_2_PO_4_, 140 mmol/L NaCl, 5.5 mmol/L glucose, 0.8 g/L BSA, pH 7.4). After centrifugation (3000 rcf, 1 min, 4°C) supernatant was removed and the RBC pellet was resolved 1:1 in SAH-buffer (HEPES buffer containing bovine serum albumin: 26.3 g/L BSA, 132 mmol/L NaCl, 4.6 mmol/L KCl, 10 mmol/L HEPES, pH 7.1).

### Percoll Density Gradient Fractionation of RBC

Fractionation of the heterogenic RBC population into different aged subpopulations was conducted *via* density gradient centrifugation using Percoll (Amersham Biosciences, Sweden). The original protocol from Bosch *et al* [52] was modified for sample amount used and the total number of different fractions received.

In detail, preparation of the different Percoll density gradients were based on Percoll stock solution with a density of 1.131 g/ml. Percoll was diluted with SAH-buffer to the desired final densities of 1.064, 1.065, 1.066, 1.068, 1.070, 1.072 and 1.076 g/ml, respectively. 2 ml of each density fraction was cautiously transferred to a 15 ml tube, starting with the densest solution. A volume of 600 μl of the prepared blood suspension was cautiously pipetted onto the preformed Percoll gradients and centrifuged at 3700 rcf for 25 min at 4°C.

The RBC layers consisting of different subpopulations were collected separately in clean tubes. The samples were washed 1:1 with GASP buffer and centrifuged (800 rcf, 10 min, 4°C). Supernatant was discarded and the pellet was washed with 1–2 ml 0.1 M phosphate buffered saline (PBS) depending on the volume of isolated fractions. After centrifugation (800 rcf, 10 min, 4°C), the supernatant was discarded. The hematocrit of the RBC pellets were adjusted to 20% and immediately used for all following analytical procedures.

### Age determination of separated RBC subpopulations

#### Morphology

Morphological changes of aged RBC subpopulations were determined microscopically using a Leica-microscope coupled to a CCD-camera (DXC-1850P, Sony, Germany). The RBC subpopulations were fixed with 2% formaldehyde (final concentration) and blood smears were prepared on microscope slides as described by Suhr *et al*. [37]. Pictures were taken after heat fixation for RBC immobilisation. Magnification for all pictures was 400-fold.

#### MCV

The mean cellular volume (MCV) of RBC subpopulations was determined in RBC suspensions with an automated haematology analyser (Sysmex KX-21N, USA).

#### Phosphatidylserine (PS) externalisation

Old and pathologically changed RBC externalise the senescence marker PS, which is preferentially bound 1:1 to the calcium-dependent phospholipid-binding protein AnnexinV [53]. AnnexinV can therefore act to determine the concentration of extracellular PS using an AnnexinV-ELISA (IBL International, Germany). After haematocrit adjustment to 20%, the measurements were performed according to the manufacturers’ instructions.

Briefly, the ELISA is based on a peroxidase dependent oxidation step followed by a colour change from blue to yellow which was detected at 450 nm. A standard curve was prepared from seven human AnnexinV standard dilutions (0, 0.78, 1.56, 3.13, 6.25, 12.5, 25, 50 ng/ml) and human AnnexinV sample concentrations were directly determined from the standard curve with linear regression. The intensity of the emitted light was proportional to the AnnexinV concentration in the sample and was analysed out of the slope of the regression line.

### RBC deformability

Deformability of the RBC-subpopulations was measured ektacytrometrically *via* the laser-optical-rotational-cell-analyser (LORCA, RR Mechatronics, Netherlands) [54,55]. The LORCA system has been described in detail elsewhere [55,56].

Briefly, 10 μl of RBC suspension with RBC of different age were solved in 2.5 ml of an isotonic 0.14 mM polyvinylpyrrolidone (PVP) solution (osmolarity 300 mOsmol*L^-1^, viscosity 28.7 mPa*sec at 37°C), respectively. 1 ml of the RBC/PVP solution was sheared in a Couette system at physiological pH 7.4 and 37°C. Nine shear rates between 0.3 and 50 Pa (0.3, 0.57, 1.08, 2.04, 3.87, 7.34, 13.92, 26.38, 50 Pa) were applied and width (W) and length (L) of the diffraction pattern was used by the LORCA software to calculate an Elongation Index (EI) EI = (L-W)/(L+W).

EI values were plotted as a Michaelis-Menten-like function and the maximal Elongation Index (EI_max_) was calculated using non-linear regression. EI_max_ describes the theoretical maximum deformability at infinite shear stress.

### Nitric oxide measurement

Nitrite represents a reliable marker for NO production for both physiological and pathological conditions [57–59].

According to the protocol of Pelletier *et al* [60] and Hendgen-Cotta *et al* [61], RBC subpopulations were immediately mixed with preservation solution (800 mM K_3_[Fe(CN)_6_], 100 mM NEM, 10 V-% Igepal, 90 V-% aqua dest.) in a 5:1 ratio and snap frozen. For measurement of RBC nitrite content, the samples were mixed with methanol in a 1:2 ratio for protein precipitation and centrifuged at 21000 rcf for 10 min at 4°C. The supernatant was collected in new reaction tubes and nitrite concentration of supernatant was determined by injecting 100 μl into an acidified tri-iodide solution that reduces nitrite to NO gas. Along with a helium gas-stream NO was purged into an ozone-based chemiluminescence NO detector (CLD 88e, EcoPhysics, Switzerland).

The Chart FIA software (EcoPhysics, Switzerland) was used to analyze the area under the curve. All samples were measured in triplicate. Using aqueous calibration solution with known nitrite concentration allowed calculation of sample nitrite content.

### Determining L-arginine concentration

Concentration of L-arginine, the substrate of both RBC-NOS and arginase I, was measured using an L-arginine-ELISA (Immundiagnostik AG, Germany). The test principle is based on the method of competitive enzyme linked immunoassays. After lysis of RBC subpopulations in a cold tempered ultrasonic bath for about 30 min, centrifugation at 21000 rcf at 4°C for 10 min and supernatant transfer in clean reaction tubes, the measurements were performed according to the manufacturers’ instructions. Detection was performed using an enzymatic colour reaction measured in a photometer at 450 nm. The inverse proportional intensity to the L-arginine concentration was analysed with a 4-parameter-algorithm with a linear ordinate for optical density and a logarithmic abscissa for concentration.

All samples were measured in duplicates and L-arginine concentration was determined from a dose response curve of absorbance unit using standard dilutions of defined concentration (0, 12.5, 30, 75, 150, 300 μM).

### Immunohistochemical staining

RBC of the different subpopulations were fixed in 2% formaldehyde (final concentration) for 20 min as described by Suhr *et al* [37]. Briefly, RBCs were dispersed on a slide and heat fixed. Afterwards, a control and a test area were marked with a grease pencil and washed twice with 1x Tris-Buffered Saline (TBS). RBC were incubated with 0.1% trypsin for 30 min at 37°C. Then the endogenous peroxidase was inhibited with 80% methanol / 5% H_2_O_2_ / 15% aqua dest for 30 min at room temperature (RT) and unspecific binding sites of the blood smear were blocked with 3% milk powder solution in 1x TBS. The primary antibody was diluted in 0.3% milk powder and 0.09% Tween20 (1:500 for Rabbit anti Human RBC-NOS Serine^1177^, Upstate, USA; AB_310608) and pipetted on the test area and incubated for 1 hour at RT. The control area was incubated with 0.3% milk powder and 0.09% Tween20 for 1 h at RT without the primary antibody. Following TBS washing and blocking of unspecific binding sites using 3% Normal Goat Serum (Dako Agilent Technologies, Denmark), both areas were incubated with the second antibody (1:400 Goat anti-rabbit antibody, Dako Agilent Technologies, Denmark; AB_2313609) displaying specifity against the first antibody, for 30 min at RT. A horseradish-peroxidase solution (Sigma Aldrich, USA), diluted 1:400 in 1x TBS, was added to the areas and a 3,3'-Diaminobenzidine solution was used for staining reaction. The stained blood smears were dehydrated in raising alcohol solutions, mounted with Entellan (Merck KGaA, Germany) and covered.

Pictures were taken using a Leica microscope coupled to a CCD-camera (DXC-1850P, Sony, Germany) with a magnification of 400-fold to determine RBC-NOS activation. Grey value determination was used for staining intensity analysis. The mean grey values of the edge of 50 RBC on at least 4 different visual fields of the test area and 10 RBC on at least 2 visual fields of the control area were measured with the software “Image J” (National Institutes of Health, USA).

Total immunostaining intensity for each fraction was calculated as the mean of measuring RBC grey value minus background grey value which was obtained on a cell free area of the slide. Mean grey values of the control field were subtracted from mean grey values of the test field to yield net grey value representing staining of the RBC [36,37].

### Enzyme activity assay: arginase assay

The protocol for determination of arginase I activity was adapted from Corraliza *et al* [62]. The hematocrit of all RBC subpopulations was adjusted to 20%.

RBC of the different subpopulations were diluted 1:10 in protein cell lysis buffer (2.0 ng/μl Leupeptin, 2.0 ng/μl Aprotinin, 1.0 mM PMSF, pH 7.5) containing protease inhibitors and detergents, which were further diluted in arginase assay buffer (50 mM Tris-HCl, 0.5% Igepal, 0.25 M NaCl, 1.0 mM Na_3_VO_4_, 0.5 mM NaF in aqua dest., pH 7.5). The samples were further diluted in a 1:2 ratio with a manganese dichloride buffer (10 mM MnCl_2_, 50 mM Tris-HCl, pH 7.5) for arginase I activation.

The solutions were incubated for 10 min at 55°C and 0.5 M L-arginine was then added and samples were incubated for another hour at 37°C under mild shaking conditions. The reaction was stopped by 1:9 dilution in an acid mix (1 V-% H_2_SO_4_: 3 V-% H_3_PO_4_: 7 V-% aqua dest) and 2:1 9% IPSF. Samples were thoroughly mixed and the tubes punctured at the lid, followed by incubation for 45 min at 95°C in the dark, 10 min at RT in the dark and final centrifugation for 5 min at 18000 rcf and RT.

Samples were measured photometrically in triplicate at 540 nm in microtiter plates. Concentrations of the samples were determined using urea standards with known concentrations (0, 100, 200, 250, 300, 350, 400, 500 nM). Concentration was calculated as nmol/μg urea production per hour.

For calculation of produced urea in dependence of individual protein concentration, protein content of the different fractions was determined using the Protein DC Kit (BioRad, Germany).

### *In vitro* approach: Influence of RBC-NOS inhibition on RBC rheology

To determine the influence of RBC-NOS inhibition and thus reduced NO content in RBC during aging, blood samples of 10 healthy subjects (mean ± SD): age [years]: 25.9 ± 5.8, height [cm]: 178.1 ± 4.8, weight [kg]: 74.5 ± 8.6) were prepared as described above. *In vitro* experiments were only performed in venous blood of healthy individuals. Blood was either incubated with PBS (-L-NIO) or with L-NIO (+L-NIO) (10 μM; Cayman Chemicals, USA) to inhibit RBC-NOS for 1 hour at 37°C in a water bath. The samples were centrifuged for 5 min at 2200 rcf at RT and the supernatant was discarded and the RBC pellets were taken to measure deformability and nitrite concentration as described above.

### Statistical Analysis

Statistical analyses of the data were performed by using statistics software package GraphPadPrism 6 (La Jolla, USA).

Gaussian distribution of the data was calculated and repeated measure analysis of variances (ANOVA) with Tukey *Post hoc* test was applied to normal distributed data. Non Gaussian distributed data were analysed by Friedman test and Dunn’s multiple comparison *Post hoc* test. Correlation and linear regression analyses were performed between RBC-NOS^Ser1177^ immunostaining and RBC nitrite concentration for both HC and DM and both R and R^2^ were presented. Statistical differences were considered to be significant for values of P < 0.05. Values of the *in vivo* investigations were compared to the respective previous density. Data from *in vitro* experiments were compared between-LNIO and +L-NIO. Descriptive statistics of the data was presented as mean ± standard deviation unless otherwise described.

## Results

### Determination of RBC aging after Percoll density gradient centrifugation

#### Proportion

**Fig 1:**
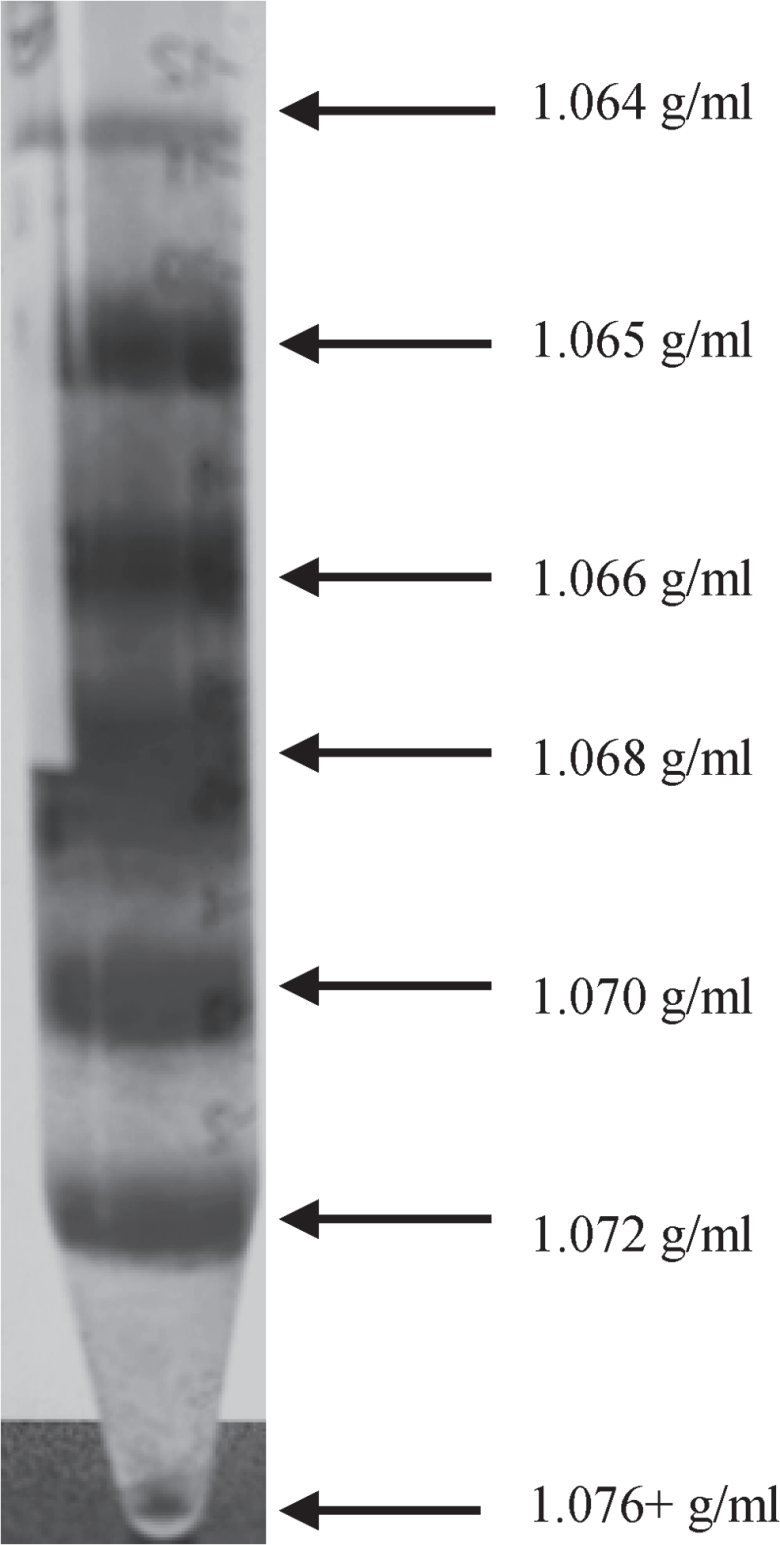
Red blood cell layers after Percoll density gradient centrifugation. Seven RBC fractions were generated by Percoll density gradient centrifugation. Least dense fraction on the top consisted of youngest RBC; increasing density indicated increasing RBC age. Arrows indicate the respective density of Percoll solution. Subpopulations were clearly separated and collected. Oldest fraction at the bottom was described as 1.076+ g/ml, with “+” indicating possible even denser RBC than the densest Percoll layer of 1.076 g/ml.

**Fig 2:**
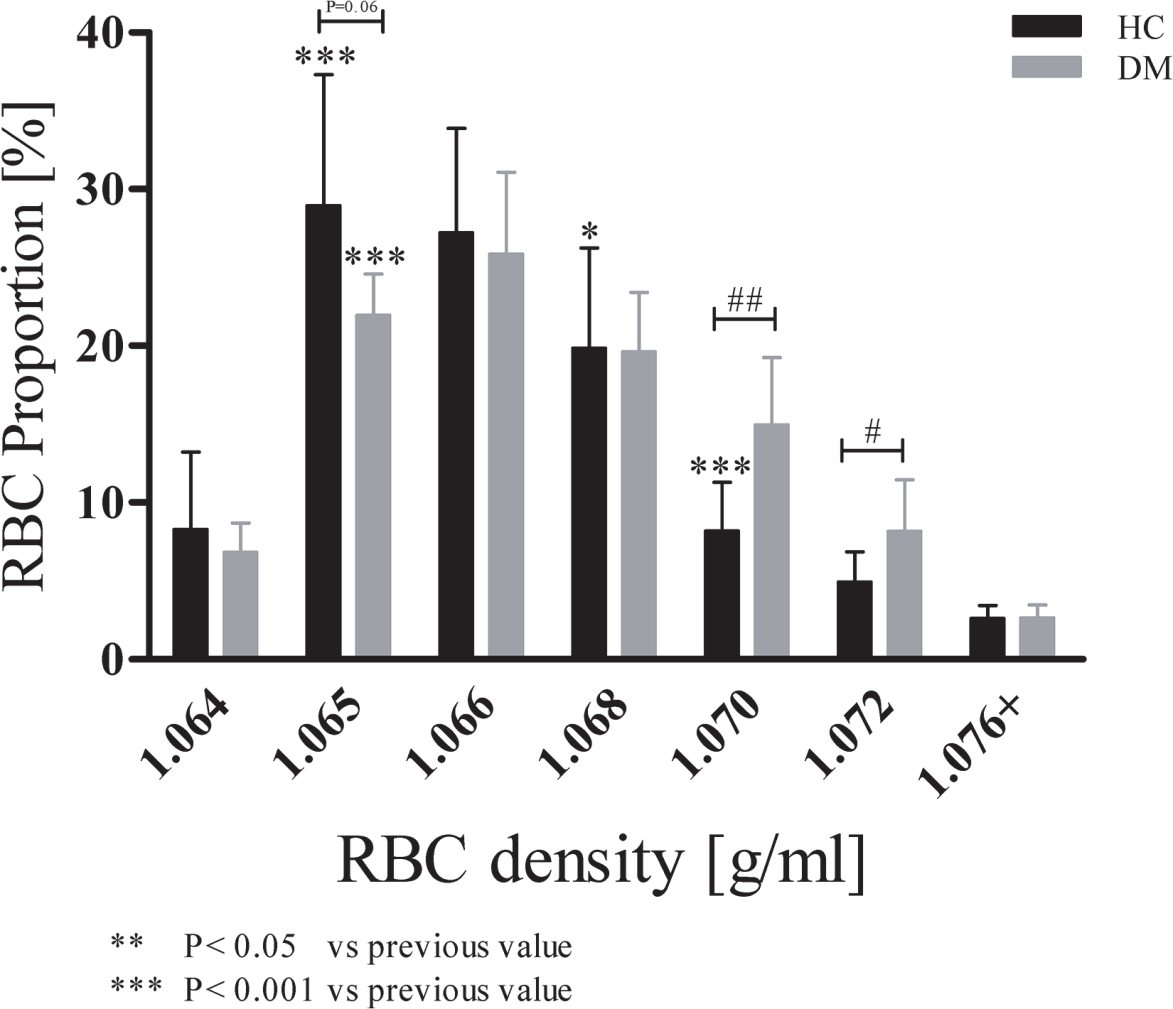
RBC proportion of HC and DM in dependence of increased cell age. Bars show RBC proportion of fractionated subpopulations to total RBC volume, which were divided into young (1.064 g/ml), middle old (1.065–1.068 g/ml) and old (1.070–1.076+ g/ml) RBC. In both study groups, middle old RBC represented the main proportion on total RBC volume. DM showed significantly higher amount of old RBC compared to HC. Data are presented as mean ± standard deviation of n = 15 (HC) and n = 4 (DM).

Seven RBC layers were received after Percoll density centrifugation (Fig 1). In HC, calculation of the proportion of each RBC subpopulation to total volume revealed that middle aged RBC with densities of 1.065–1.068 g/ml represented 76.0 ± 1.1% of total RBC volume while the portion of young RBC was calculated to be 6.8 ± 4.9% and the old fractions including RBC with densities of 1.070–1.076+ g/ml constituted 15.7 ± 1.2%. For DM, portion of young, middle aged and old RBC was calculated to be 6.7 ± 1.9%, 67.4 ± 1.3% and 25.8 ± 1.8% (P < 0.01 compared to HC) (Fig 2).

#### Morphology

**Fig 3:**
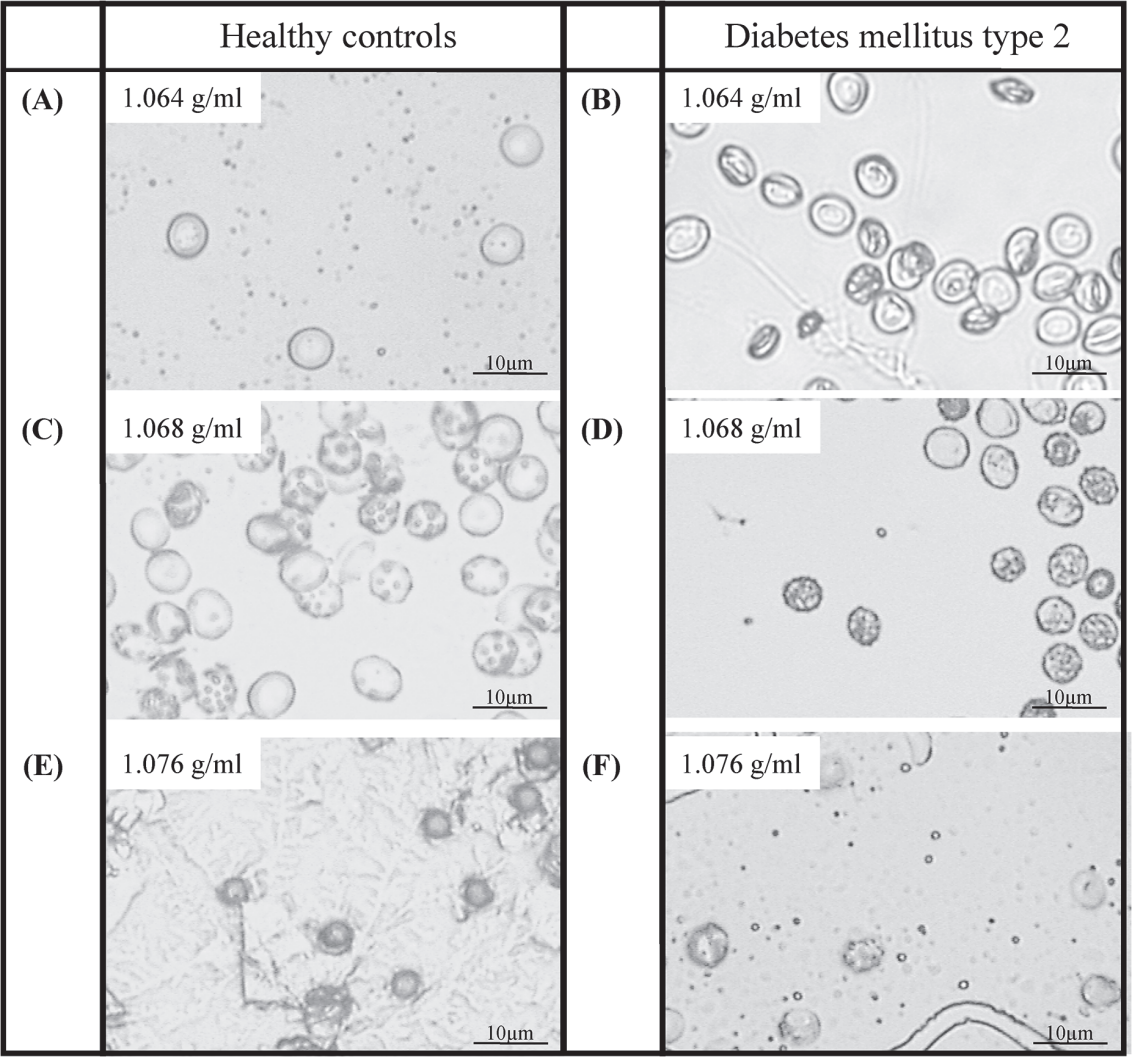
Representative microscopic pictures of the seven isolated RBC subpopulations. Density of the RBC increased with increasing cell age, and cell shape changed from discoid to echinocyte. In RBC of DM, cell shape changes were more pronounced. Magnification for all pictures was 400-fold.

Morphological changes of RBC during aging as presented in Fig 3 showed changes in size and shape in both RBC of HC and DM. Common changes observed in both groups were that young RBC (density 1.064 g/ml) exhibited a uniform spherical morphological shape, whereas the middle aged fractions (represented by density 1.068 g/ml) had in part a more squat, spherocyte shape. In addition, there was an increase in membrane evagination indicated through spots on the membrane surface which hints to membrane vesiculation. Further morphological changes were observed in old subpopulations (represented by density 1.076+ g/ml). Membrane vesiculation was further enhanced and cell shape changed to echinocyte. RBC of DM showed enhanced morphological changes with smaller and more shapeless RBC throughout the different fractions in comparison with healthy controls.

#### MCV

**Fig 4:**
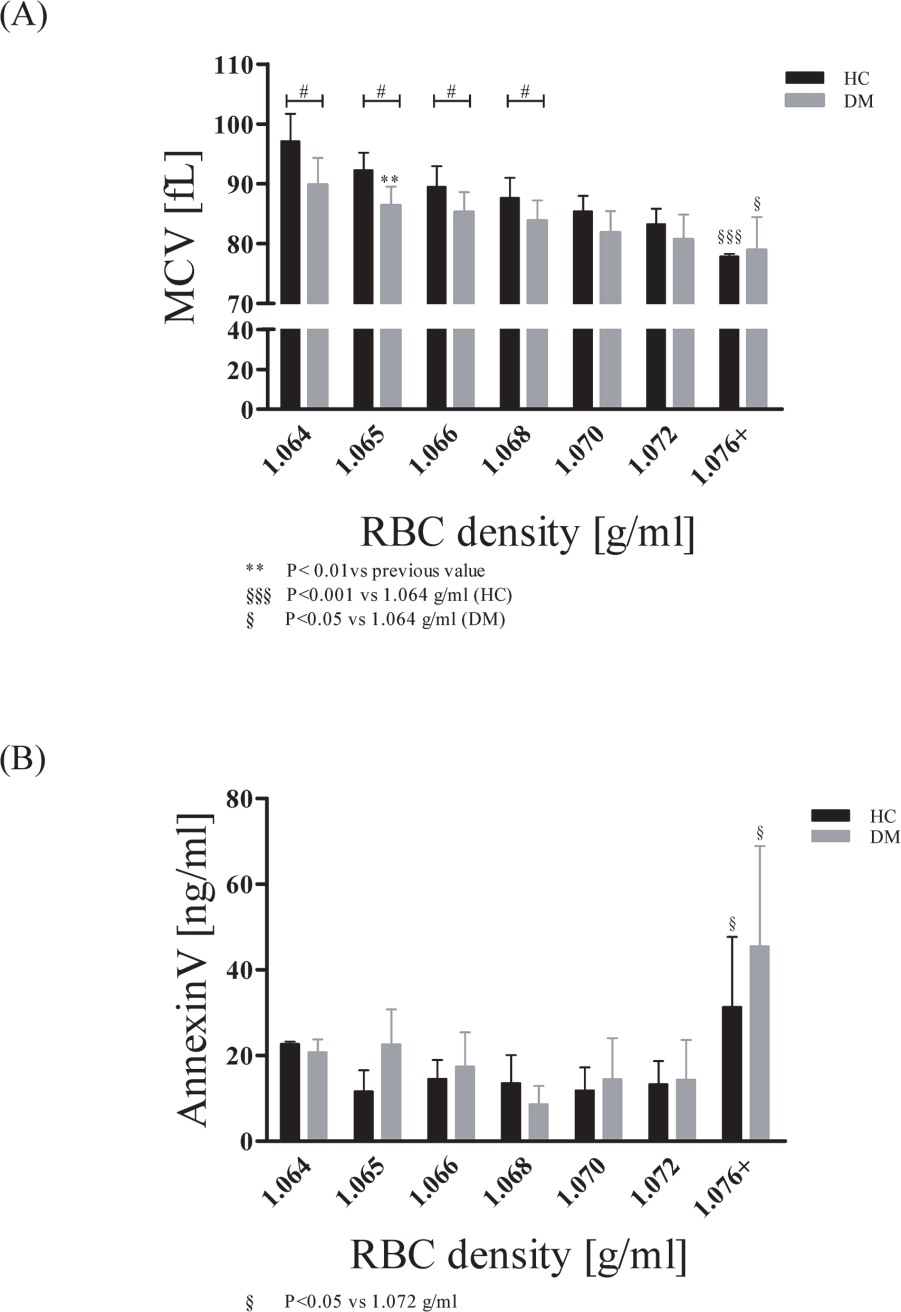
Mean Cellular Volume (MCV) and phosphatidylserine (PS) externalisation with increasing cell age in HC and DM. (A) Mean cellular volume constantly decreased during aging in both groups. MCV of young and middle aged RBC was significantly decreased in DM compared to HC. RBC of HC lost 20% of MCV during aging (P < 0.001; comparison of old and young RBC). RBC of DM lost 12% of MCV during aging (P < 0.05; comparison of old and young RBC). Data are presented as mean ± standard deviation of n = 15 (HC) and n = 4 (DM). (B) In both groups, Annexin V, a marker for PS externalisation, significantly increased in the oldest subpopulation (P < 0.05 compared to 1.072 g/ml). Data are presented as mean ± standard deviation of n = 4 (HC) and n = 4 (DM).

In HC, MCV significantly decreased by 19.9 ± 6.3% (P < 0.001) during aging and in DM, MCV significantly decreased by 12.1 ± 3.7% during aging (P < 0.05). In DM, MCV was significantly lower in young and middle aged RBC (1.064–1.068g/ml) compared to MCV of HC (Fig 4A).

#### Annexin V/ PS externalisation

Annexin V, a marker for externalized PS, was predominantly found on the oldest RBC subfractions (Fig 4B). PS concentration on the outer membrane surface increased from 1.064 g/ml to 1.076+ g/ml fraction by 38.3 ± 7.4% (P < 0.05) in HC and by 119.6 ± 11.9% (P < 0.05) in DM.

### Rheology and metabolite determination: Deformability and nitrite as sensitive markers

#### RBC deformability

**Fig 5:**
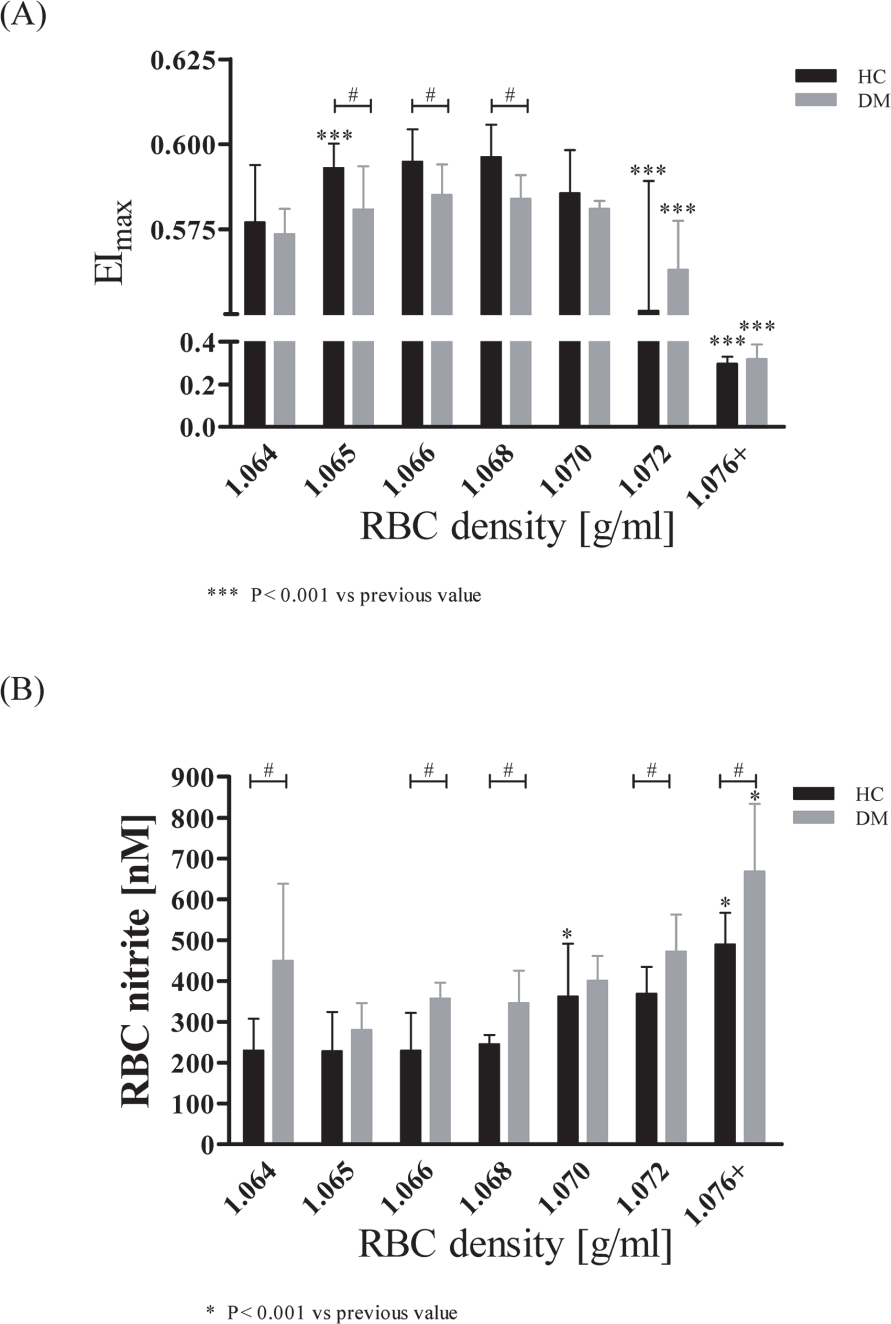
Maximal RBC deformability (EI_max_) and nitrite concentration in RBC of HC and DM during cell aging. (A) In RBC of HC, EI_**max**_ values increased from 1.064 g/ml to 1.065 g/ml (P < 0.001), remained constant to 1.068 g/ml and then decreased with increasing cell age. Oldest RBC (1.076+ g/ml) showed lowest EI_**max**_ (P < 0.001 compared to 1.072 g/ml). EI_**max**_ of middle aged RBC (1.065–1.068 g/ml) was significantly lower in DM compared to HC. EI_**max**_ of DM significantly decreased during aging. Data are presented as mean ± standard deviation of n = 15 (HC) and n = 4 (DM). (B) In HC, RBC nitrite concentration remained constant from 1.064 g/ml to 1.068 g/ml and then significantly increased reaching its maximum values at 1.076+ g/ml. RBC nitrite concentration was significantly higher in DM compared to HC for 1.064, 1.066, 1.0681.072 and 1.076+ g/ml. RBC nitrite concentration in RBC of DM increased during aging. Data are presented as mean ± standard deviation of n = 5 (HC) and n = 4 (DM).

In HC, maximum deformability EI_max_ significantly increased from fraction 1.064 g/ml to fraction 1.065 g/ml (P < 0.001). Highest EI_max_ values were calculated for the middle aged fractions (1.065–1.068 g/ml) and EI_max_ then significantly decreased with increasing RBC age (1.072 g/ml: P < 0.001 compared to 1.070 g/ml; 1.076+ g/ml: P < 0.001 compared to 1.072 g/ml). In DM, EI_max_ significantly decreased with aging with lowest EI_max_ in old RBC (1.072 g/ml: P < 0.001 compared to 1.070 g/ml; 1.076+ g/ml: P < 0.001 compared to 1.072 g/ml). EI_max_ of middle aged RBC was significantly lower in DM compared to HC (Fig 5A). (For single elongation indices obtained from all nine shear rates see S1 and S2 Tables).

#### RBC nitrite concentration

In HC, RBC nitrite concentration remained constant for densities of 1.064–1.068 g/ml and then significantly increased from 1.068 g/ml to 1.070 g/ml (P < 0.05) and from 1.072 g/ml to 1.076+ g/ml (P < 0.05). In DM, RBC showed higher RBC nitrite concentration compared to HC and RBC nitrite significantly increased with increasing cell age (Fig 5B).

### Enzymatic properties—immunostaining and enzymatic assays for RBC-NOS and arginase I

#### RBC-NOS activation

**Fig 6:**
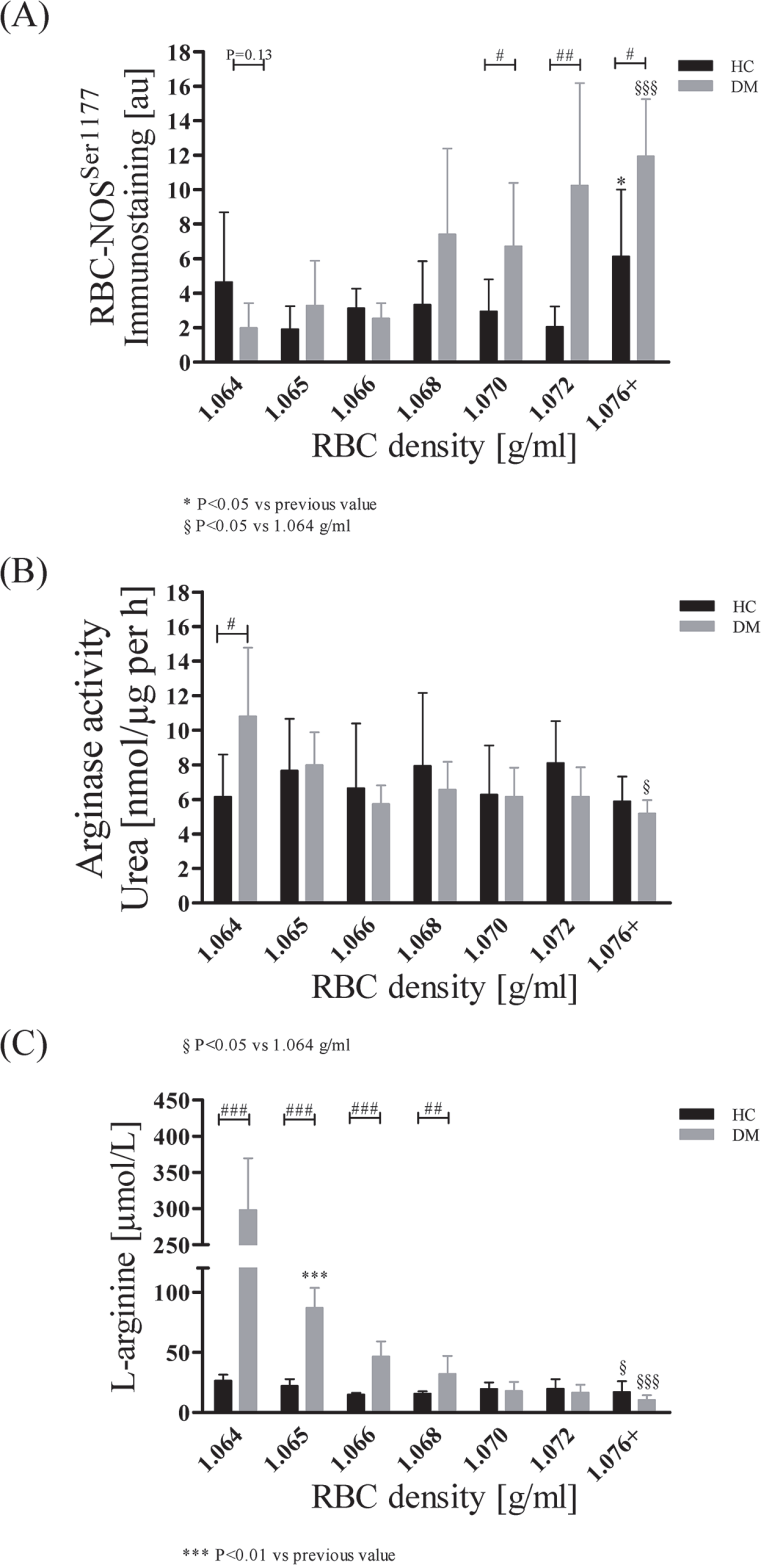
RBC-NOS activation, arginase I activity and L-arginine concentration in RBC of HC and DM during increasing cell age. (A) In HC, RBC-NOS phosphorylation at its Serine^1177^ residue, representing activation of the enzyme, was significantly increased in subpopulation 1.076+ g/ml (P < 0.05 compared to 1.072 g/ml). In DM, RBC-NOS^Ser1177^ significantly increased during aging with significantly higher values obtained in DM compared to HC in RBC fractions 1.070, 1.072 and 1.076+ g/ml. Data are presented as mean ± standard deviation of n = 8 (HC) and n = 4 (DM). (B) Arginase I activity was represented by the synthesis of the arginase I product urea. In both groups, arginase activity remained constant in all measured subpopulations. Data are presented as mean ± standard deviation of n = 4 (HC) and n = 4 (DM). (C) In HC, L-arginine concentration remained unchanged during aging. DM showed significantly higher L-arginine values compared to HC for young and middle old RBC (1.064–1.068 g/ml). L-arginine concentration of DM decreased with increasing cell age. Data are presented as mean ± standard deviation of n = 8 (HC) and n = 4 (DM).

In HC, activation of RBC-NOS remained unchanged throughout the subfractions 1.064–1.072 g/ml, but RBC-NOS phosphorylation of Serine^1177^ significantly increased in the oldest subpopulation (1.076+ g/ml) (P < 0.05 compared to 1.072 g/ml). In DM, RBC-NOS phosphorylation significantly increased during aging (P < 0.001 comparing fractions 1.064 and 1.076+ g/ml). RBC-NOS phosphorylation of young RBC was slightly decreased in DM (P = 0.13 versus HC) but was significantly higher in fractions 1.070 (P < 0.05), 1.072 (P < 0.01) and 1.076+ g/ml (P < 0.05) compared to HC (Fig 6A).

#### Arginase I activity

In HC, arginase I activity did not significantly change during aging. In young RBC (1.064 g/ml) arginase I activity was significantly higher in DM compared to HC (P < 0.05). Arginase I activity of DM decreased with increasing RBC age (P < 0.05 comparing 1.076+ with 1.064 g/ml) (Fig 6B).

#### RBC L-arginine concentration

In HC, L-arginine concentration significantly decreased from young to old RBC (P < 0.05: 1.076+ versus 1.064 g/ml). DM showed significantly higher L-arginine levels in young and middle aged RBC compared to HC. L-arginine concentration of RBC from DM decreases with increasing RBC age (P < 0.001: 1.076+ versus 1.064 g/ml) (Fig 6C).

#### Correlation/Linear regression analyses

**Fig 7:**
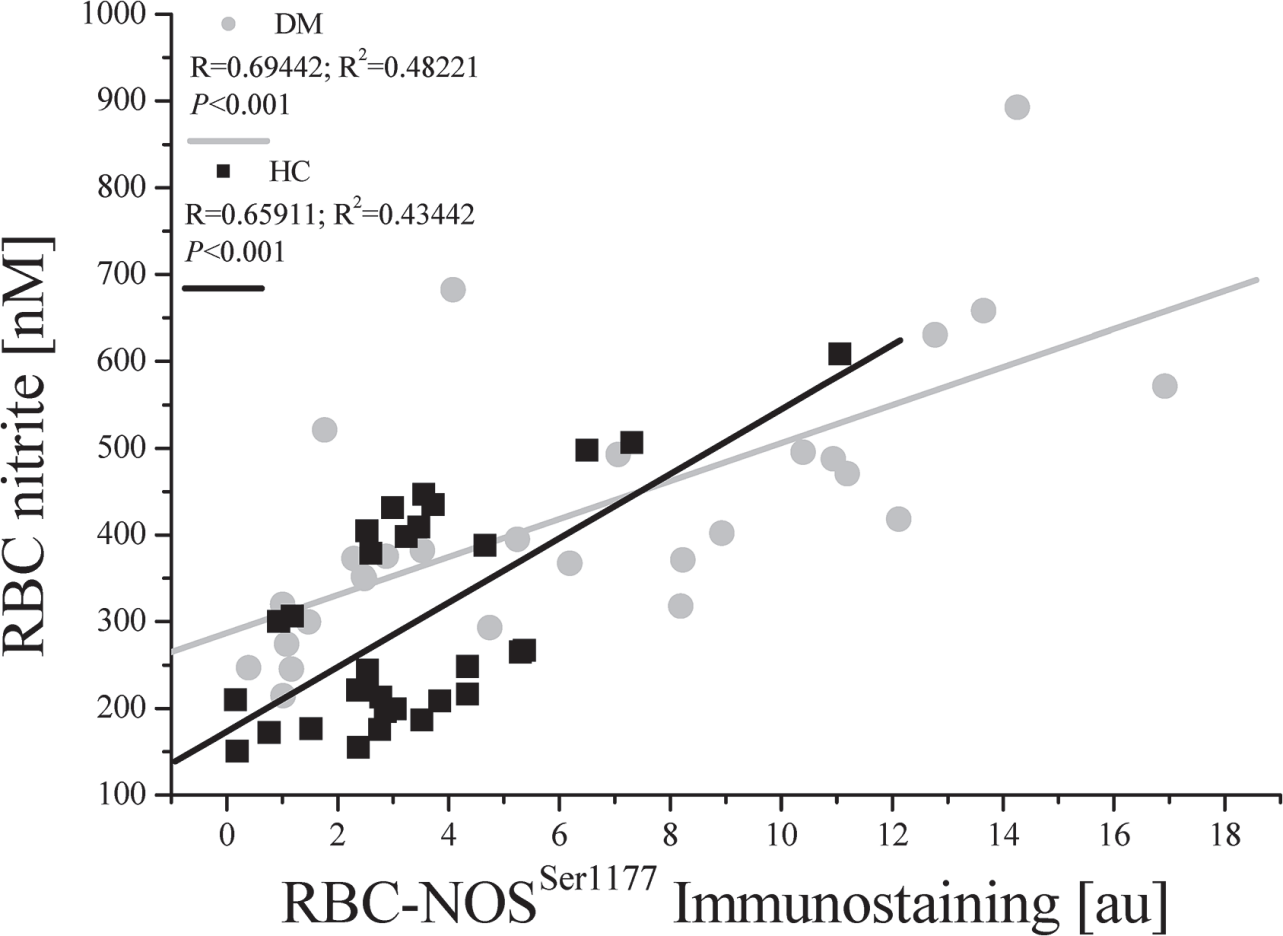
Linear regression of RBC-NOS^Ser1177^ immunostaining and RBC nitrite concentration in HC and DM. Calculation of R and R^2^ (goodness of fit) revealed positive correlation of RBC-NOS^Ser1177^ and RBC nitrite for both study groups.

Correlation and regression analyses revealed a strong positive relation of RBC-NOS^Ser1177^ and RBC nitrite in HC and DM (Fig 7), respectively.

### Influence of RBC-NOS inhibition by L-NIO on NO production and deformability

**Fig 8:**
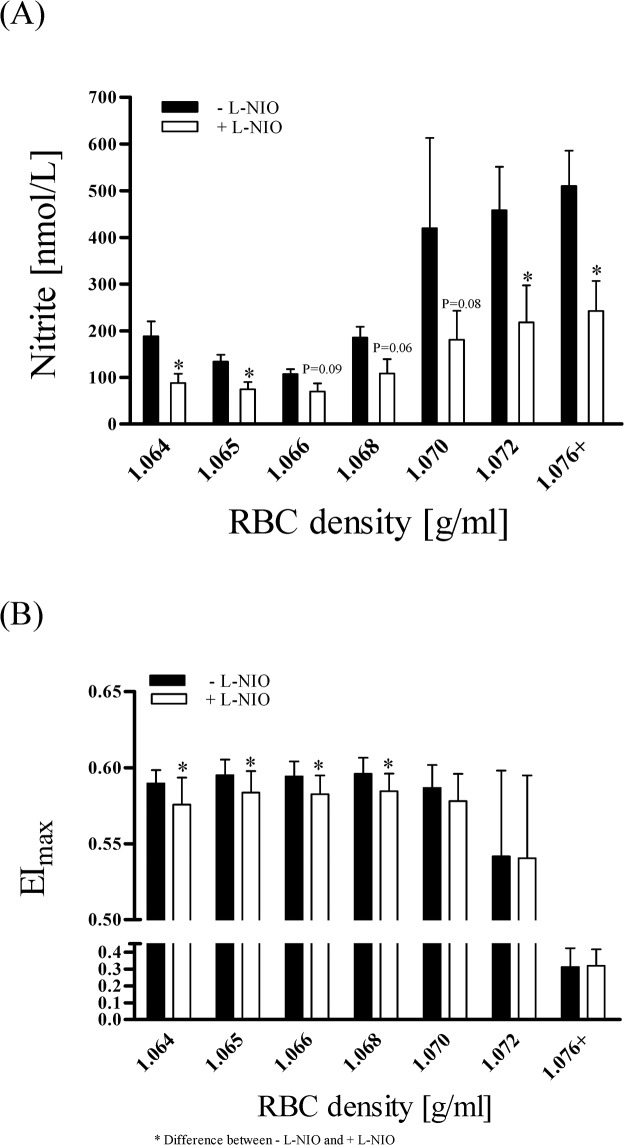
Nitrite concentration and maximum RBC deformability (EI_max_) in RBC after L-NIO incubation in comparison to untreated RBC. (A) RBC nitrite concentration was decreased in all subpopulations by L-NIO. Data are presented as Mean ± Standard Error of MEAN (n = 10). (B) EI_**max**_ values significantly decreased in subpopulations of 1.064 g/ml to 1.068 g/ml after L-NIO incubation. No decrease was observed in old subpopulations (1.070 g/ml and 1.076+ g/ml). Data are presented as Mean ± Standard Deviation (n = 10).

To provide information about the inhibitory potential and sensitivity of the different RBC fractions to NO inhibition, RBC-NOS activation was directly inhibited using L-NIO. The determination of nitrite concentration showed significantly lower nitrite levels after inhibition with L-NIO. Nitrite concentration of young and old RBC was reduced by approximately 54% by L-NIO while the nitrite concentration was reduced by approximately 40% in middle aged RBC (Fig 8A). EI_max_ of RBC subfractions ranging from 1.064–1.068 g/ml was significantly decreased after L-NIO incubation. No changes in EI_max_ were observed in RBC subfractions of 1.070–1.076+ g/ml after L-NIO incubation (Fig 8B).

## Discussion

RBCs are a very heterogeneous cell population exhibiting different rheologic and enzymatic properties. RBC have been demonstrated to carry a functional NO synthase which produces NO [12] and it was previously reported that RBC-NOS produced NO plays a crucial role in RBC deformability [12,36,39]. RBC aging was associated to a series of molecular events that finally lead to cell clearance but whether changes in RBC NO synthesis during *in vivo* aging may relate these events is unknown so far. RBC of healthy controls and patients with type 2 diabetes mellitus were thus separated according to age and *in vivo* RBC-NOS activation, RBC deformability, RBC nitrite, respective NO, concentration, activity of RBC-NOS competitor arginase I and substrate bioavailability of both enzymes during the aging process were examined. Additional *in vitro* experiments were performed with blood of healthy individuals to examine the influence of specific RBC-NOS inhibition on rheologic properties and RBC nitrite, respective NO concentration in the different aged RBC subpopulations.

The results of the present study revealed decreasing RBC deformability, increasing nitrite concentration and increasing RBC-NOS activation during aging. These changes were observed in both healthy controls and patients with type 2 diabetes mellitus, but more pronounced in type 2 diabetics. Young RBC of patients with type 2 diabetes mellitus showed highest L-arginine concentration, highest arginase I activity and least RBC-NOS activation. The course of the parameters reversed during aging. Comparable changes of arginase I activity and L-arginine concentration were not observed in healthy controls. RBC-NOS inhibition decreased nitrite concentration in all subpopulations while RBC deformability of old RBC was not affected.

### Percoll density gradient centrifugation is suitable to separate RBC by age

Separation of blood by Percoll density gradient centrifugation yielded in seven clearly distinguishable RBC layers. Although Gifford *et al* questions the reliability and accuracy of RBC fractionation according to cell age with density gradient centrifugation [63], the multiple age determination parameters including MCV and AnnexinV/PS externalization performed in this work prove fractionation according to cell age.

Subdividing the seven fractions into young (1.064 g/ml), middle (1.065–1.068 g/ml) and old RBC (1.070–1.076+ g/ml) revealed that DM had less middle aged and more old RBC compared to HC. These data underline findings made by Handelman and Levin [64], and Mazzanti *et al* [65] who reported a reduced mean life span and faster senescence of RBC from patients with type 2 diabetes mellitus.

Morphology of RBC changed during aging. Whereas the youngest RBC showed nearly round and unaffected cell shape, older RBC became smaller and echonocytic. The observed changes on the outer surface indicated membrane evagination [66]. Due to the missing nucleus of mature RBC, the predefined enzymatic repertoire requires waste decomposition *via* membrane exocytosis [66]. For instance, the main RBC protein hemoglobin becomes glycated and carbamylated with aging, leading to accumulation of denatured hemoglobin at the inner membrane site [9]. Microvesiculation is energetically more favourable in comparison to the whole new synthesis of otherwise functional RBC, but leads to permanent loss of membrane bilayer parts, cell ingredients and cellular water [52]. Data presented in this study prove decreasing MCV with increasing cell age. Before clearance of the oldest RBC from the circulation, approximately 20% of the volume is lost in HC. These observations indicate the approximate vesiculation capacity of normal RBC, which could be a limiting factor for RBC life time. MCV of RBC from DM was significantly lower compared to HC and the vesiculation capacity was markedly reduced (12%), which obviously is a limiting factor for RBC life span [67]. Once vesiculation capacity is exhausted, old RBC generate senescence signals recognised by macrophages. These signals include the expression of neoantigenic regions in the membrane domain, which results in binding of autologous IgG or the externalisation of phosphatidylserine on the outer membrane surface [9]. In both groups, HC and DM, PS externalisation was determined on the outside of the oldest RBC fraction with PS concentrations being twice as high as on the outside of young RBC. An increased capacity for membrane vesiculation could favor the prevention of oxidative stress and early RBC hemolysis, which has been described for cardiovascular diseases like diabetes [68,69]. Decreased RBC volume is associated with RBC rigidity and impaired RBC deformability thus increasing the risk for cardiovascular events [69,70]. A shift to older RBC as observed in DM may eventually explain impaired cardio- and microvascular function associated with this disease [16,71].

### NO signaling in RBC is altered during RBC aging

It was previously reported that older RBC show reduced deformability, diminished capacity to release ATP in response to mechanical deformation [70] and accelerated NO-dioxygenation rates compared to younger RBC [72]. This may reduce NO bioavailability, diminish vasodilatation and oxygen supply. Although the proportion of old RBC was relatively small in comparison to the main middle aged fraction, it is assumed that 20% of old RBC would increase overall inhibitory potential of NO dependent vasodilatation in all RBC by ~ 8% [72]. These *in vitro* observed kinetics indicate that small changes in the population proportion may have significant effects on vascular homeostasis and NO-signaling. Therefore it seems of considerable interest to shift the RBC proportion to the younger ones, especially in disease states accompanied with insufficient oxygen supply and circulatory disorders.

#### RBC deformability changes during RBC aging

RBC deformability depends on a variety of cellular properties: surface to volume ratio, intracellular calcium concentration, activation of calcium ATPase, sodium-potassium ATPase activation, pH or messengers like prostaglandins and NO [73].

Impaired deformability is reported for many diseases including diabetes mellitus [41] and results in diminished oxygen supply to the microvascular system [36]. In DM, deformability was significantly lower in middle aged RBC in comparison to HC. In both groups, deformability decreased with increasing cell age with lowest EI_max_ values measured in the oldest RBC. Interestingly, in HC the youngest RBC did not exhibit the highest EI_max_, possibly due to highest MCV observed in this group. The high volume capacity has an adverse effect on rheological properties. This subpopulation may possess too much cellular volume to squeeze fast and suitable through the capillaries. Further membrane remodelling and volume reduction may occur during aging which cause the increase in RBC deformability observed between 1.064 and 1.065 g/ml.

#### RBC-NOS dependent NO production increased during RBC aging

NO produced in RBC is oxidised to nitrite in the presence of molecular oxygen [23,29,30]. Nitrite thereby acts as a storage pool for NO and it was shown that nitrite represents a sensitive marker for NO production and bioavailability [74]. RBC nitrite concentration, and thus NO level, increased during aging with DM showing higher nitrite values compared to HC. Higher RBC nitrite concentrations were previously confirmed in whole blood of type 2 diabetics [75]. High nitrite levels may counteract rheological restrictions and decreasing MCV during the aging process. It can be assumed that without the enhanced NO concentration, deformability of old RBC would be even more diminished. Among others, similar results were published by Smith *et al* [6], Linderkamp *et al* [66] and Bosman *et al* [67]. This was in part confirmed by the presented *in vitro* results as inhibition of NO synthesis further decreased RBC deformability in young and middle aged RBC. Old RBC also showed reduced nitrite content after L-NIO application but RBC deformability was not further affected.

In RBC, NO is enzymatically produced by RBC-NOS with phosphorylation of RBC-NOS on its serine 1177 representing activation of the enzyme [37]. RBC-NOS^Ser1177^ was increased in senescent RBC in both healthy subjects and patients with type 2 diabetes mellitus. These results matched the observation of increasing nitrite level in old RBC reaching its maximum in the oldest fraction. Interestingly, in old RBC phosphorylation of RBC-NOS^Ser1177^ was significantly higher in DM compared to HC. Correlation analyses exhibited that nitrite and thus NO concentration depends on RBC-NOS^Ser1177^ in DM and HC. These results indicate that increased RBC nitrite concentration was caused by increased RBC-NOS activation to partly counteract decreased deformability. But this compensation seemed to be insufficient for the oldest RBC subpopulation. It is suggested that the NO produced is incapable to bind to the RBC cytoskeleton to regulate deformability. It was recently shown that microparticles formed by vesiculation contained a functional endothelial NOS which still had enzymatic activity and were found to produce NO [76]. Furthermore, RBC are one of the major vesicle-secreting cells [77]. Thus it is speculated that the permanent formation of vesicles, as observed microscopically in aging RBC, may have led to the embedding of membrane associated RBC-NOS in old RBC. RBC-NOS produced NO was still detected but could not exert its biological function of improving RBC deformability. The experimental evidence that enhanced NO synthesis leads to modifications of α- and β-spectrins at the cytoskeleton of RBC, leading to improved deformability, was recently shown and is one of the most important binding sites of regulatory NO in RBC [36]. These modifications were induced by direct activation of RBC-NOS and subsequent NO production. Separation of the cytoskeleton from the active RBC-NOS by microvesicles may interrupt efficient NO transport for compensation. These findings suggest NO as possible pharmacological target to improve deformability and life span of RBC.

#### Activity of RBC-NOS competitor arginase and concentration of common substrate L-arginine decrease during RBC aging

Due to its common substrate L-arginine and coexpression in a variety of different cell tissues, arginase I and NOS interdependences were intensively examined in the past decade [49]. Arginase I is known to play an important role in the regulation of the immune system and pathological development as it negatively regulates NO synthesis [78,79]. Different cardiovascular disease states are described which exhibit decreased NO availability and increased arginase activity such as hypertension and diabetes [47,79–81]. Increased arginase I activity was found in young RBC from type 2 diabetics and activity decreased with increasing cell age. In parallel, highest L-arginine concentrations were found in young RBC and decreased with increasing cell age. Considering the low RBC-NOS activation in young RBC, compared to HC, the data confirm a shift to arginase I activity at least in young RBC from DM. A similar correlation was not observed in middle aged or old RBC and no such correlation was observed in RBC from HC. The data of the presented study thus did not confirm previous observations of increased arginase activation in diabetic rats [81] but comparable studies in humans are lacking.

In conclusion, *in vivo* RBC aging results in shape changes and changes in enzyme activities which finally reduce RBC deformability in both health and disease with a higher prevalence in the disease state. The inability of high NO levels in old RBC to affect cell function point out that RBC-NOS may be isolated which might prevent NO from binding to the cytoskeleton, necessary to affect RBC deformability. The data underline the importance of NO bioavailability to extent cell life and to maintain healthy rheologic characteristics which also positively affect blood flow through the microcirculation and oxygen supply to the surrounding tissues and organs. The results obtained in this study could contribute to the development of new therapeutic strategies to improve rheological properties in patients with cardiovascular diseases by increasing RBC NO bioavailability.

## Supporting Information

S1 Table Single elongation indices obtained for all tested shear rates and red blood cell fractions in healthy controls (HC). Data are presented as mean ± standard deviation of n = 15 (HC). Statistical differences were calculated for the respective previous group and are marked with * for P < 0.05; ** for P < 0.01 and *** for P < 0.001.
(DOCX)

S2 Table Single elongation indices obtained for all tested shear rates and red blood cell fractions in patients with type 2 diabetes (DM). Data are presented as mean ± standard deviation of n = 4 (DM). Statistical differences were calculated for the respective previous group and are marked with * for P < 0.05; ** for P < 0.01 and *** for P < 0.001. Differences between the HC and DM were marked with # for P < 0.05; ## for P < 0.01 and ### for P < 0.001.
(DOCX)
