# Supplementary material for: Increase in Red Blood Cell-Nitric Oxide Synthase Dependent Nitric Oxide Production during Red Blood Cell Aging in Health and Disease: A Study on Age Dependent Changes of Rheologic and Enzymatic Properties in Red Blood Cells
Source: PLoS One. 2015 Apr 22;10(4):e0125206. doi: 10.1371/journal.pone.0125206 (PMC4406474; doi:10.1371/journal.pone.0125206)
Supplement: S1 Table — Data are presented as mean ± standard deviation of n = 15 (HC). Statistical differences were calculated for the respective previous group and are marked with * for P < 0.05; ** for P < 0.01 and *** for P < 0.001. (DOCX) [file pone.0125206.s001.docx]

**S 1 Table: Single elongation indices obtained for all tested shear rates and red blood cell fractions in healthy controls (HC).**

| Percoll density | 1.064 g/ml | 1.065 g/ml | 1.066 g/ml | 1.068 g/ml | 1.070 g/ml | 1.072 g/ml | 1.076+ g/ml |
| --- | --- | --- | --- | --- | --- | --- | --- |
| Shear rate [Pa] |  |  |  |  |  |  |  |
| 0.3 | 0.155 ± 0.012 | 0.160 ± 0.018 | 0.146 ± 0.010  ** | 0.138 ± 0.008  * | 0.114 ± 0.009  *** | 0.095 ± 0.018  *** | 0.072 ± 0.007 |
| 0.57 | 0.189 ± 0.012 | 0.175 ± 0.011  ** | 0.161 ± 0.009  *** | 0.148 ± 0.005  *** | 0.133 ± 0.005  *** | 0.109 ± 0.015  *** | 0.076 ± 0.007  * |
| 1.08 | 0.263 ± 0.014 | 0.251 ± 0.012  ** | 0.232 ± 0.013  *** | 0.214 ± 0.010  *** | 0.187 ± 0.012  *** | 0.149 ± 0.023  *** | 0.076 ± 0.002  ** |
| 2.04 | 0.352 ± 0.012 | 0.344 ± 0.011 | 0.327 ± 0.013  *** | 0.307 ± 0.011  *** | 0.276 ± 0.015  *** | 0.227 ± 0.036  *** | 0.112 ± 0.003  * |
| 3.87 | 0.430 ± 0.014 | 0.427 ± 0.007 | 0.415 ± 0.011  *** | 0.398 ± 0.011  *** | 0.366 ± 0.014  *** | 0.311 ± 0.044  *** | 0.166 ± 0.006  * |
| 7.34 | 0.486 ± 0.015 | 0.492 ± 0.005  * | 0.484 ± 0.009  *** | 0.474 ± 0.008  *** | 0.446 ± 0.014  *** | 0.388 ± 0.053  *** | 0.205 ± 0.011  * |
| 13.92 | 0.524 ± 0.015 | 0.533 ± 0.003  * | 0.529 ± 0.009  * | 0.525 ± 0.008  * | 0.505 ± 0.013  *** | 0.445 ± 0.063  ** | 0.229 ± 0.020  * |
| 26.38 | 0.556 ± 0.016 | 0.568 ± 0.004  ** | 0.564 ± 0.009  * | 0.561 ± 0.007  * | 0.544 ± 0.012  *** | 0.488 ± 0.066  ** | 0.257 ± 0.020  * |
| 50 | 0.581 ± 0.016 | 0.595 ± 0.005  ** | 0.591 ± 0.010  * | 0.586 ± 0.008  * | 0.568 ± 0.012  *** | 0.515 ± 0.066  ** | 0.284 ± 0.015  ** |

Data are presented as mean ± standard deviation of n=15 (HC). Statistical differences were calculated for the respective previous group and are marked with * for P < 0.05; ** for P < 0.01 and *** for P < 0.001.
