# Supplementary material for: Increase in Red Blood Cell-Nitric Oxide Synthase Dependent Nitric Oxide Production during Red Blood Cell Aging in Health and Disease: A Study on Age Dependent Changes of Rheologic and Enzymatic Properties in Red Blood Cells
Source: PLoS One. 2015 Apr 22;10(4):e0125206. doi: 10.1371/journal.pone.0125206 (PMC4406474; doi:10.1371/journal.pone.0125206)
Supplement: S2 Table — Data are presented as mean ± standard deviation of n = 4 (DM). Statistical differences were calculated for the respective previous group and are marked with * for P < 0.05; ** for P < 0.01 and *** for P < 0.001. Differences between the HC and DM were marked with # for P < 0.05; ## for P < 0.01 and ### for P < 0.001. (DOCX) [file pone.0125206.s002.docx]

**S 2 Table: Single elongation indices obtained for all tested shear rates and red blood cell fractions in patients with type 2 diabetes (DM).**

| Percoll density | 1.064 g/ml | 1.065 g/ml | 1.066 g/ml | 1.068 g/ml | 1.070 g/ml | 1.072 g/ml | 1.076+ g/ml |
| --- | --- | --- | --- | --- | --- | --- | --- |
| Shear rate [Pa] |  |  |  |  |  |  |  |
| 0.3 | 0.195 ± 0.022  ### | 0.208 ± 0.032  ### | 0.189 ± 0.024  *  ### | 0.185 ± 0.032  ### | 0.160 ± 0.011  ### | 0.126 ± 0.013  **  ## | 0.078 ± 0.010  *** |
| 0.57 | 0.177 ± 0.011 | 0.173 ± 0.015 | 0.168 ± 0.011 | 0.160 ± 0.010  ## | 0.145 ± 0.007  **  ## | 0.133 ± 0.005  **  ## | 0.095 ± 0.008  ***  # |
| 1.08 | 0.252 ± 0.016 | 0.243 ± 0.015  * | 0.239 ± 0.008 | 0.225 ± 0.010  *  # | 0.199 ± 0.012  ***  # | 0.176 ± 0.010  **  # | 0.110 ± 0.010  ***  ## |
| 2.04 | 0.342 ± 0.013 | 0.336 ± 0.012  * | 0.332 ± 0.009 | 0.316 ± 0.009  * | 0.290 ± 0.013  ** | 0.254 ± 0.011  *** | 0.146 ± 0.023  ** |
| 3.87 | 0.423 ± 0.006 | 0.421 ± 0.006 | 0.417 ± 0.005  * | 0.405 ± 0.007 | 0.379 ± 0.012  ** | 0.342 ± 0.009  ** | 0.194 ± 0.034  ** |
| 7.34 | 0.479 ± 0.005 | 0.483 ± 0.005  ## | 0.481 ± 0.005 | 0.475 ± 0.004 | 0.456 ± 0.006  ** | 0.420 ± 0.010  ** | 0.240 ± 0.043  *** |
| 13.92 | 0.521 ± 0.005 | 0.526 ± 0.007  ## | 0.526 ± 0.004 | 0.523 ± 0.002 | 0.509 ± 0.004  ** | 0.481 ± 0.013  ** | 0.273 ± 0.050  *** |
| 26.38 | 0.556 ± 0.005 | 0.561 ± 0.006  *  # | 0.563 ± 0.004 | 0.559 ± 0.002 | 0.549 ± 0.003  ** | 0.524 ± 0.012  * | 0.306 ± 0.058  ** |
| 50 | 0.583 ± 0.006 | 0.591 ± 0.004  * | 0.592 ± 0.001 | 0.586 ± 0.004  * | 0.574 ± 0.006  ** | 0.552 ± 0.012  * | 0.321 ± 0.062 ** |

Data are presented as mean ± standard deviation of n=4 (DM). Statistical differences were calculated for the respective previous group and are marked with * for P<0.05; ** for P<0.01 and *** for P<0.001. Differences between the HC and DM were marked with # for P<0.05; ## for P<0.01 and ### for P<0.001.
